# Los comités de ética de investigación en la Ciudad Autónoma de Buenos Aires: a catorce años de la implementación de la Ley 3301

**DOI:** 10.18294/sc.2023.4482

**Published:** 2023-10-30

**Authors:** Cecilia Quattrucci, Andrés Martín Pereira, Agustina L. Galletti, Luciana B. Scolaro, Lucila A. Pastori, Verónica M. Sandez, Fabio O. Barceló

**Affiliations:** 1 Especialista en Evaluación de Políticas Públicas. Maestranda en Investigación en Ciencias Sociales, Facultad de Ciencias Sociales, Universidad de Buenos Aires, Ciudad Autónoma de Buenos Aires, Argentina. ceciliaquattrucci@gmail.com Universidad de Buenos Aires Facultad de Ciencias Sociales Universidad de Buenos Aires Ciudad Autónoma de Buenos Aires Argentina ceciliaquattrucci@gmail.com; 2 Antropólogo, Especialista en Epidemiología. Instructor de Residentes en Investigación en Salud, Ministerio de Salud de la Ciudad de Buenos Aires, Ciudad Autónoma de Buenos Aires, Argentina. andres.m.pereira@gmail.com Ministerio de Salud de la Ciudad de Buenos Aires Ministerio de Salud de la Ciudad de Buenos Aires Ciudad Autónoma de Buenos Aires Argentina andres.m.pereira@gmail.com; 3 Profesora en Ciencias Antropológicas, Residente de Investigación en Salud, Ministerio de Salud de la Ciudad de Buenos Aires, Ciudad Autónoma de Buenos Aires, Argentina. aggalletti@gmail.com Ministerio de Salud de la Ciudad de Buenos Aires Ministerio de Salud de la Ciudad de Buenos Aires Ciudad Autónoma de Buenos Aires Argentina aggalletti@gmail.com; 4 Licenciada en Nutrición. Residente de Investigación en Salud, Ministerio de Salud de la Ciudad de Buenos Aires, Ciudad Autónoma de Buenos Aires, Argentina. lucianabelenscolaro@gmail.com Ministerio de Salud de la Ciudad de Buenos Aires Ministerio de Salud de la Ciudad de Buenos Aires Ciudad Autónoma de Buenos Aires Argentina lucianabelenscolaro@gmail.com; 5 Licenciada en Ciencias Antropológicas, Residente de Investigación en Salud, Ministerio de Salud de la Ciudad de Buenos Aires, Ciudad Autónoma de Buenos Aires, Argentina. lucilapastori@gmail.com Ministerio de Salud de la Ciudad de Buenos Aires Ministerio de Salud de la Ciudad de Buenos Aires Ciudad Autónoma de Buenos Aires Argentina lucilapastori@gmail.com; 6 Profesora en Ciencias Antropológicas, Residente de Investigación en Salud, Ministerio de Salud de la Ciudad de Buenos Aires, Ciudad Autónoma de Buenos Aires, Argentina. mverosandez@gmail.com Ministerio de Salud de la Ciudad de Buenos Aires Ministerio de Salud de la Ciudad de Buenos Aires Ciudad Autónoma de Buenos Aires Argentina mverosandez@gmail.com; 7 Especialista en Estado, Gobierno y Democracia. Residente de Investigación en Salud, Ministerio de Salud de la Ciudad de Buenos Aires, Ciudad Autónoma de Buenos Aires, Argentina. fabo.barcelo@gmail.com Ministerio de Salud de la Ciudad de Buenos Aires Ministerio de Salud de la Ciudad de Buenos Aires Ciudad Autónoma de Buenos Aires Argentina fabo.barcelo@gmail.com

**Keywords:** Ética en Investigación, Comités de Ética en Investigación, Investigación Cualitativa, Sistemas de Salud, Argentina, Research Ethics, Research Ethics Committees, Qualitative Research, Health Systems, Argentina

## Abstract

El presente artículo se propone analizar el proceso de implementación de los comités de ética en investigación en la Ciudad Autónoma de Buenos Aires, cuya conformación se observa en el marco de un proceso histórico de implementación de políticas de investigación en salud a nivel nacional y jurisdiccional. Desde un enfoque de investigación cualitativo, observacional y de corte transversal, se realizó un relevamiento de fuentes secundarias de información pública, y entrevistas semiestructuradas y en profundidad a integrantes de los comités de la Ciudad Autónoma de Buenos Aires. Los resultados se presentan agrupados en tres ejes: 1) la conformación de los comités de ética en investigación; 2) procesos de trabajo, haciendo hincapié en el impacto de la pandemia de covid-19; y 3) obstáculos y propuestas, que se focaliza en las mejoras identificadas por sus integrantes.

## Abstract (en)

This article analyzes the implementation of research ethics committees in the Autonomous City of Buenos Aires, a process that took place in the context of a historical process of implementing health research policies at both the national and jurisdictional levels. Employing a qualitative, observational, and cross-sectional approach, we gathered secondary sources of public information and conducted semi-structured, in-depth interviews with members of the committees in the Autonomous City of Buenos Aires. The results are presented in relation to three aspects: 1) the composition of the research ethics committees; 2) work processes, emphasizing the impact of the COVID-19 pandemic; and 3) obstacles and proposals, that focuses on improvements identified by committee members.

## INTRODUCCIÓN

En el campo de la ética de la investigación en salud, se reconoce una larga y nutrida trayectoria de reflexiones y debates en torno a las pautas éticas y normativas que regulan la investigación en salud a nivel mundial. La historia de los documentos internacionales sobre ética ha sido la respuesta a distintas violaciones de los derechos humanos de las personas participantes de las investigaciones. Aún con la existencia de códigos y declaraciones, se requieren estrictos controles y seguimiento, tanto gubernamentales como de cuerpos independientes locales, regionales e internacionales, que evalúen los aspectos éticos de la investigación en salud; tarea en la cual resultan fundamentales los comités de ética de investigación (CEI)^1^. 

Los documentos sobre ética en investigaciones en salud más relevantes son la Declaración de Helsinki de 2013, promulgada por la Asociación Médica Mundial, y las *Pautas éticas internacionales para la investigación relacionada con la salud con seres humanos*, del año 2016, elaboradas por el Consejo de Organizaciones Internacionales de las Ciencias Médicas (CIOMS) en colaboración con la Organización Mundial de la Salud (OMS). Además, existen legislaciones que interpretan las normas internacionales a la luz de los contextos locales: a nivel nacional, la Resolución 1480/2011 Guía para Investigación en Seres Humanos, del Ministerio de Salud de la Nación; y en la Ciudad Autónoma de Buenos Aires (CABA) se destaca la Ley 3301 del año 2009 sobre Protección de derechos de sujetos en investigaciones en salud. 

El proceso de conformación de los CEI en la CABA puede observarse en el marco de un proceso histórico de implementación de políticas de investigación en salud a nivel nacional y jurisdiccional^2^. La Argentina sigue a nivel nacional la política de investigación para la salud aprobada por la OMS y la Organización Panamericana de la Salud (OPS) que, desde el año 2000, se propone el fortalecimiento de los sistemas nacionales de investigación en salud para toda Latinoamérica, definidos como “el conjunto de actores que gobiernan, gestionan, coordinan, requieren, producen, comunican o utilizan la investigación y sus resultados para promover, restablecer, mejorar o mantener el estado de salud y desarrollo de una población”^3^. Estos sistemas nacionales se inscriben en un proceso de desarrollo desigual de la investigación en salud en Latinoamérica y se caracterizan por:

…la carencia de políticas nacionales y de marcos regulatorios y normativas en aspectos específicos, dispersión de los sectores u organismos clave del sistema de investigación, obstáculos para coordinar acciones entre los distintos actores involucrados, limitaciones para establecer mecanismos adecuados y estandarizados aptos para determinar las necesidades y prioridades, entre otros.^3^

En el caso de la CABA, la Ley 3301 responde a un vacío legal preexistente y da inicio formal a la conformación de los CEI, con el fin de garantizar la protección de los derechos de las personas participantes. En este sentido, la ley señala que

...la inclusión de seres humanos en investigaciones sólo podrá realizarse después de que la investigación haya sido revisada y aprobada por el CEI acreditado y competente, y con la constancia de una voluntad de sujeto manifestada a través del proceso de consentimiento libre y esclarecido.^4^

En la misma línea, se especifican los roles, las funciones, la composición, entre otras características de los CEI que forman parte del sistema de salud, incluyendo tanto al subsector público como privado. Asimismo, se señala la tarea de evaluar los riesgos y beneficios para los sujetos de las investigaciones, además de garantizar las condiciones que deben cumplir los equipos de investigación. Como parte del mismo proceso de conformación, la Ley 3301 crea el Comité Central de Ética en Investigaciones como espacio consultivo y educativo de discusión y reflexión.

En línea con lo anterior, se han realizado estudios comparativos y sobre el funcionamiento de los CEI en Argentina, que incluyen a la CABA como jurisdicción, que permiten identificar características comunes y obstáculos en su proceso de conformación^5^^,^^6^^,^^7^. En esta dirección, el presente artículo se propone presentar un análisis parcial de los resultados cualitativos de un proyecto de investigación en curso titulado: “La conformación de los CEI: debates e hitos en el sistema público de salud de la Ciudad de Buenos Aires desde el 2009-2022”, el cual se desarrolla en el marco de la Residencia Posbásica de Investigación en Salud del Ministerio de Salud de la CABA y cuenta con la aprobación del CEI del Hospital General de Agudos Dr. Abel Zubizarreta. Entre sus objetivos se encuentra la descripción del proceso de conformación de los CEI en efectores de salud públicos de la CABA. Se indaga sobre las particularidades del proceso de implementación en cada efector de salud, partiendo del supuesto de que obedecen a una historia específica y presentan efectos en las condiciones para el desarrollo de las investigaciones y sus mecanismos regulatorios. 

El análisis aquí propuesto contribuirá al debate sobre las características que han adquirido los CEI y las investigaciones en salud en la CABA en el período estudiado (2009-2022). 

## MÉTODOS

El proyecto que da origen a este artículo utiliza un enfoque de investigación cualitativo, observacional y de corte transversal. La investigación se desarrolló durante los años 2021 y 2022 (años en los cuales se realizó el análisis documental y la realización de entrevistas), sin embargo, el período bajo análisis comprendió los años 2009 a 2022. 

El diseño metodológico implementado en este proyecto es “flexible”^8^. Este concepto alude a la posibilidad de advertir durante la investigación emergentes y nuevos ejes con los cuales profundizar en la realización del trabajo de campo y la conformación de la muestra. En este sentido, el proceso de producción de los datos no se desarrolla de manera lineal, sino en forma de espiral. Este proceso reflexivo y circular permite la reelaboración de los instrumentos de construcción de los datos, a partir del análisis parcial de resultados durante el proceso de investigación.

Dentro de este marco, la investigación se implementó de forma secuencial y en tres etapas. La primera consistió en el relevamiento y análisis cuantitativo de fuentes secundarias de información pública sobre las características de los CEI en CABA. Para ello, se utilizó la información que publica el Comité Central de Ética en Investigaciones^9^ que permitió, entre otros puntos, identificar cuáles son los efectores de salud del subsector público que cuentan con un CEI en funcionamiento. También se revisó el marco normativo vigente de la CABA en lo relativo a investigación en salud^4^^,^^10^^,^^11^^,^^12^.

En la segunda etapa, mediante un muestreo intencional no probabilístico, se contactaron a referentes de distintos CEI y se realizaron, hasta el momento, seis entrevistas semiestructuradas y en profundidad^13^. En ellas se indagó sobre las experiencias de participación desde la implementación de la ley hasta el presente; las particularidades de sus procesos de conformación en el tiempo; las características de su organización interna actual; y la percepción de principales obstáculos en la práctica cotidiana. 

Para la conformación de la muestra se utilizó la técnica de bola de nieve y un criterio de diversidad. En este punto, el diseño flexible permite a la luz del análisis parcial y teórico de los datos obtenidos incluir nuevas categorías y replantear la conformación de la muestra^8^. 

Las entrevistas, realizadas a seis personas referentes de distintos CEI de la CABA, fueron desgrabadas y analizadas utilizando una matriz de análisis. En todos los casos se obtuvo el consentimiento informado de las personas participantes. En el discurso de las personas entrevistadas se identificaron puntos de saturación y elementos divergentes que permitieron caracterizar en profundidad contextos y sentidos comunes del funcionamiento de los CEI en la CABA^13^. 

En una tercera etapa, se revisaron los ejes de las entrevistas que permitieron, por un lado, incorporar los emergentes identificados en el discurso de las personas entrevistadas y, por el otro, desarrollar en profundidad la discusión teórica sobre los datos construidos. El análisis de los primeros resultados alcanzados, se agruparon en tres ejes: 1) *la conformación de los comités de ética en investigación*; 2) *procesos de trabajo*, haciendo hincapié en el impacto de la pandemia de covid-19; y 3) *obstáculos y propuestas*, que se focaliza en las mejoras identificadas por los propios integrantes de los CEI.

Con respecto al período de estudio, si bien las tres etapas señaladas no logran abordar con exhaustividad las experiencias del período de más de 20 años, sí permiten identificar elementos para orientar la indagación y ajustar la estrategia cualitativa de conformación de la muestra.

Para la presentación de los resultados, de este análisis parcial, se optó por identificar a todas las personas como integrantes de un CEI de la CABA sin diferenciar efector de salud, ni profesión. Esto se decide en parte para garantizar la confidencialidad de los datos y la información recibida. Al ser una muestra cualitativa, en un contexto institucional pequeño se utiliza esta estrategia para evitar que las personas que participaron del estudio puedan ser identificadas. 

## RESULTADOS Y ANÁLISIS

### La conformación de los comités de ética en investigación

En el texto de la Ley 3301 se menciona el carácter multidisciplinario que deben tener los CEI como organismos encargados de la evaluación ética de los proyectos de investigación. Sin embargo, no se especifican las disciplinas o especialidades del equipo de salud requeridas para las evaluaciones éticas y metodológicas. Con excepción del requerimiento de al menos una persona dedicada a la abogacía, la metodología de la investigación y la investigación médica, no se especifica como requisito otras disciplinas del equipo de salud. 

En la misma línea, otro tipo de regulaciones que se mencionan en el texto de la Ley 3301 hacen referencia a su composición en términos de cantidad de integrantes. En esta dirección, se establece como requisito al menos un 30% de personas de un mismo sexo y, además, la presencia de al menos un miembro de la comunidad ajeno al equipo de salud. 

**Figura 1:**
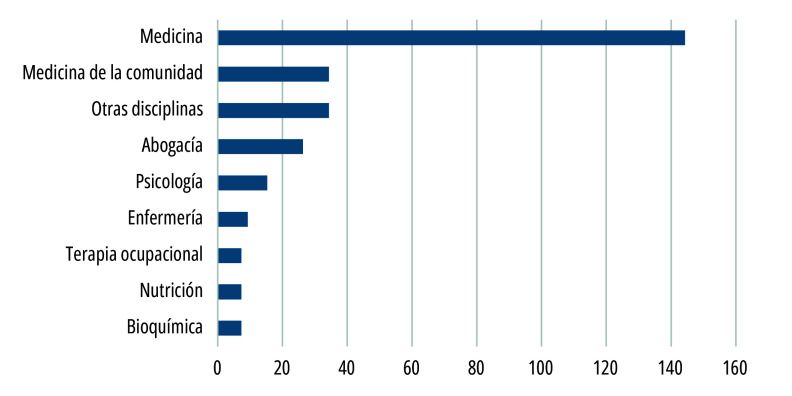
Disciplinas registradas de integrantes de los comités de ética en investigación (n=283) del subsector público de salud de la Ciudad Autónoma de Buenos Aires, Argentina. Año 2021.

Al realizar un análisis cuantitativo de la composición de los CEI, solo fueron incluidos aquellos comités del subsector público de la CABA, y se excluyeron los del subsector privado y pertenecientes a universidades. En este universo, en el año 2021 se identificaron 23 hospitales que cuentan con CEI públicos acreditados. El número total de miembros en ese período fue de 283. Al analizar su composición disciplinar (Figura 1), se observó una marcada tendencia de representación de la medicina como disciplina mayoritaria con el 51% (n= 144). La mayor representatividad de médicos y médicas en su conformación fue señalada previamente en la investigación de Sabio y Bortz^7^. Esta tendencia introduce el interrogante sobre cuáles son las perspectivas dominantes en la tarea de evaluación de los proyectos. 

Fuente: Elaboración propia con base en información publicada por el Comité Central de Ética en Investigación (CCE)

En términos teóricos, las evaluaciones éticas implican tanto la determinación del valor social de las investigaciones como de su valor científico^14^. Estos dos valores se presentan entrelazados e implican tener en consideración cuáles son las necesidades de salud para una comunidad, qué información novedosa puede aportar el estudio y si los enfoques propuestos son los apropiados para obtenerla. En este sentido, los debates metodológicos se interrelacionan con los dilemas éticos que surgen de la respuesta a los siguientes interrogantes: ¿sobre qué investigar?, ¿cómo hacerlo?, ¿para quién?^15^. Esto conlleva la tarea de adentrarse a conocer los contextos en los que los estudios tienen lugar, reconociendo la complejidad del entramado social y los diferentes intereses, necesidades y disputas de los distintos actores que intervienen. En el escenario específico de CABA, siguiendo a Candal^16^, estas consideraciones implican el reconocimiento de un contexto de desarrollo desigual, segregación socio-espacial, fragmentación y exclusión social, así como una marcada disparidad entre zonas urbanas y un proceso de modernización selectiva del espacio. Se reconoce así que es una tarea compleja y que, como tal, requiere de la intervención de una multiplicidad de miradas, que va más allá de la presencia de diferentes disciplinas en la composición de un CEI. 

En esa línea, el informe EULABOR ha señalado la escasa participación de la sociedad civil en el sistema de evaluación ética en investigación en Argentina^16^, lo que genera una falta de reflexión y debate en el funcionamiento de los comités por la ausencia de integrantes de distintos perfiles de evaluadores. Además, Candal da cuenta de la necesidad de construir una propuesta que promueva la participación de la población en los debates éticos en un contexto donde la actividad de los CEI es llevada a cabo casi íntegramente por profesionales especializados en el ámbito de la salud. 

A partir de la implementación de la Ley 3301, se instaura el carácter multidisciplinario de los CEI; sin embargo, la no especificación de las disciplinas competentes contribuye a la persistencia de la falta de representatividad de las distintas profesiones del campo de la salud. Asimismo, si bien se incluye el requisito de contar al menos con un miembro de la comunidad, el carácter inespecífico del término lleva a una heterogeneidad de personas incluidas bajo esta figura:

*En general, al miembro de la comunidad se lo entendió como alguien que no tenía una profesión de las habituales dentro de las esperadas* [...] *el miembro de la comunidad, siempre está dicho, pero es muy difícil de definir* [...] *se habla también de miembro de la comunidad como paciente… si en este hospital se trabaja mucho en temas de cáncer, a lo mejor trabajar con los miembros de esas asociaciones que trabajan e incorporan a algún miembro de esas asociaciones… mucho más difícil es poder ver un miembro de la comunidad en lo urbano que abarque por ejemplo, en el caso nuestro, las 600 manzanas.* (Integrante No. 1 de un CEI)

En las voces de otras personas entrevistadas se refleja esta falta de problematización acerca del miembro de la comunidad, entendido como alguien que no tiene una profesión “*de las habituales*” o como miembro externo, ilustrando la variabilidad en la que esta figura es considerada:

IP: *El miembro de la comunidad* [...]*, ¿cómo lo eligieron?*

E: *En realidad, es un jubilado del hospital.* IP: *Es decir, conoce la dinámica del hospital…* *E: Conoce el ambiente, conoce el hospital. Exacto.* (IP: investigador principal; E: Integrante No. 2 de un CEI)

*Los miembros de la comunidad, desde que yo entré al comité han sido siempre los mismos, los miembros externos, no de la comunidad, sino por ahí hay miembros que han venido de otros lados, si, ha ido cambiando un poco, ahora tenemos una, por ejemplo, licenciada en educación que antes no estaba, pero más o menos siempre ha habido un núcleo que la mayoría son médicos*. (Integrante No. 3 de un CEI)

Lejos de considerar que la lectura del contexto social y de las necesidades de la población es una tarea exclusiva de algunas disciplinas o roles puntales en los equipos, la heterogeneidad en la conformación de los CEI enriquece el desarrollo de esta tarea, pudiendo complejizar la lectura de las realidades que las investigaciones develan y las posibles aplicaciones e implicaciones del saber producido^15^. En este sentido, el rol del miembro de la comunidad resulta un papel central, no solo en lo relativo a la evaluación de riesgos y beneficios de la participación de poblaciones en situación de vulnerabilidad, sino también en el compromiso de garantizar que el desarrollo de investigaciones responda a necesidades de la población.

### Procesos de trabajo

Además del carácter multidisciplinario, y de acuerdo con la legislación local mencionada anteriormente, los CEI deben tener un perfil multisectorial en su composición y un balance en edad y sexo de sus integrantes, sujeto a renovación periódica. Estos requisitos deben ser plasmados por cada comité en un documento en el que se establecen sus funciones, tareas y procedimientos internos denominado “procedimientos operativos estándar” (POES).

Si bien a partir de las entrevistas realizadas se encuentran similitudes en los POES de los distintos CEI -como, por ejemplo, en relación con los mecanismos para la evaluación de proyectos y la toma de decisiones- también se identifican particularidades a lo largo de las entrevistas. En este sentido, se menciona que un criterio utilizado de distribución de las tareas recae en la trayectoria y en la disciplina de base de sus integrantes en el equipo de salud:

*La expertise tenía más que ver con lo disciplinar y, a veces, cuestiones como metodológicas donde la presidente o la secretaria decían: “Esto está bueno que lo vea el integrante que está especializado en metodología de la investigación más cuanti”. Había otra pediatra que también tenía cierta formación en metodología. Cuando se veía algo que metodológicamente no cerraba, si era algo más de índole estadística, era alguno de ellos dos quienes más lo miraban. Y después cuestiones más cualitativas, seguro que una de las personas que lo veía era yo y si era de pediatría, y si hay una pediatra, lo veía también ella.* (Integrante No. 4 de un CEI)

*Cuando agarro un protocolo que habla del mecanismo de acción de una droga, tampoco entiendo mucho, pero tengo un colega médico y un médico metodólogo con los cuales armamos el conocimiento suficiente. Bueno, entonces solo puedo hacer buenas preguntas.* (Integrante No. 5 de un CEI)

En otros casos existe la figura de “evaluación primaria”, que desarrolla una revisión exhaustiva de los protocolos a evaluar según su especialidad y que luego es puesta en común con el resto de los miembros, de manera tal que puedan evacuar dudas y realizar apreciaciones.

*En general, tratan de darle al especialista, o sea, no me dan a mí uno de farmacia como evaluador primario. Después, todos vemos todo, pero, digamos, se designa como evaluador primario a aquel que tiene un conocimiento más especializado.* (Integrante No. 2 de un CEI)

Sin embargo, más allá de estos procedimientos en la tarea de evaluación de proyectos, en muchos de los casos, la disponibilidad de tiempo se configura como el principal criterio para la distribución de estas tareas. En algunas de las entrevistas se presenta como determinante, dado que se enfatiza de forma significativa sobre la recarga de trabajo y al tiempo destinado a tareas administrativas. En la misma línea, desde la perspectiva de las personas entrevistadas, se destaca que el tiempo asignado dentro de sus áreas de trabajo para la realización de las tareas del CEI es muy escaso. Lo que usualmente es denominado por las personas entrevistadas como “tiempo protegido” no es suficiente para abordar las reuniones, lecturas de protocolos, acompañamiento de los investigadores, divulgación, etc. De esta forma, en la organización de los tiempos de trabajo hospitalarios subyace un orden en el que la evaluación de proyectos de investigación no es una tarea priorizada. En consecuencia, se observa que muchas de las personas integrantes de los CEI terminan de realizar sus labores fuera del horario laboral destinado para ello y en sus casas. En esta dirección, las tareas vinculadas a la investigación sanitaria, en tanto realizadas por el personal de salud, no son ajenas a la prevalencia de *burnout* descrita en la literatura y en aumento en el contexto de la pandemia^17^:

*…*él que tiene un poco más de tiempo dice*: “Yo lo miro”.* (Integrante No. 2 de un CEI)

*…Dos horas es la reunión… pero hay mucho otro laburo que no se ve y que lo hago en casa o en los ratos libres que me queda en el consultorio. Pero es lo que tenemos todos formalmente, todos los miembros del hospital tenemos autorizadas dos horas, que nos sirve, como te digo, exclusivamente para las reuniones.* (Integrante No. 3 de un CEI)

Por otro lado, con relación a las modificaciones que trajo la pandemia de covid-19, en las entrevistas resaltan los cambios que se introdujeron durante el período de aislamiento social preventivo y obligatorio en cuanto al uso de tecnologías de la información y la comunicación (TIC), que comenzaron a funcionar como nuevo parámetro para la organización del trabajo en los CEI:

*…Aún antes de la pandemia, como tenemos muchos integrantes externos, la reunión en persona ya era bastante difícil, pero desde que entramos en pandemia, las reuniones fueron siempre a través de Zoom y, muchas veces, la mayoría de las veces, dependiendo de la disponibilidad de los otros, en horario o fuera del horario de nuestro trabajo habitual. Es un CEI que, por lo menos, dos veces al mes se reúne seguro y, a veces, una vez por semana. Generalmente se ponen dos o tres fechas posibles y en base a la decisión de la mayoría, se define un horario.* (Integrante No. 5 de un CEI)

A lo largo de las entrevistas el período de la pandemia se presenta como un parteaguas, entre un antes y un después y, una vez finalizada, muchas de las estrategias virtuales utilizadas durante ese periodo continuaron vigentes, permitiendo flexibilizar la relación entre espacios y la distribución de tiempos para desarrollar las actividades.

*Bueno…, la pandemia nos cambió. Si, nos reuníamos presencialmente hasta antes de la pandemia. Como tuvimos que reacreditar, hace un año, menos de un año, al reacreditar teníamos que enviar nuestros POE.... aprovechamos ahí para actualizarlos y en nuestros nuevos POE, en los actuales, está permitida la reunión virtual, nosotros la habilitamos* [...] *Ustedes saben que hay miembros externos en todo CEI, hay miembros externos, miembros de la comunidad, bueno... los que viven cerca vienen. Muchos han tenido que vencer su temor a contagiarse, pero están viniendo. Pero algunos que viven un poco más lejos a veces vienen, a veces no.* (Integrante No. 3 de un CEI)

Sin embargo, más allá del carácter disruptivo de esta eventualidad y su consecuente reestructuración en la organización del trabajo en salud, la pandemia tuvo otras repercusiones en el funcionamiento de los CEI. Durante este periodo se incrementaron los estudios vinculados al covid-19, en los cuales se resaltaba el carácter de urgencia que la situación exigía. En este sentido, se expidieron disposiciones y recomendaciones desde el Ministerio de Salud para regular el trabajo de los CEI que, si bien recomendaban instancias de revisión expeditivas para proyectos de investigación, resaltaron que no se debía disminuir la rigurosidad en el análisis ético y metodológico durante su evaluación^18^.

*Empezamos a funcionar online, matamos todo el papel y todo pasó a electrónico. Hacíamos reuniones semanales en vez de quincenales y eran híbridas. La gente que no es del hospital que no venga, quédense en la casa… compremos una camarita, la ponemos ahí y re funciona todo eso. La tecnología fue un aporte y el resto te diría… humano. Lo que pasó entre los miembros del comité estuvo buenísimo.* (Integrante No. 5 de un CEI)

*La presidenta y la secretaria estuvieron trabajando con cierta urgencia en la evaluación de los protocolos que tuvieran que ver con el plasma y a todas las cosas que se estaban investigando en relación a la pandemia, como hacer los consentimientos en esos casos, etc. Algunas de las actividades tomaron el carácter de urgencia que antes no tenían. Así que eso también fue un cambio en relación a los tiempos de expedición de dictámenes. Básicamente eso, dificultad para la reunión y urgencia de esos tiempos* [...] *en relación a la cantidad de proyectos, no sentí que se potenció…* (Integrante No. 2 de un CEI)

En líneas generales, a lo largo de las entrevistas, la percepción de los cambios introducidos en el contexto de la pandemia de covid-19 produjo efectos diferentes en los CEI. Mientras en algunos se menciona que pudieron aprovechar el contexto y específicamente a partir del uso de TIC fortalecer la dinámica de trabajo, en otros casos, no se observó tan claramente lo potenciador de la situación. En este último punto, las referencias en el discurso de las personas entrevistadas también señalaron la urgencia como un fuerte obstáculo en ese contexto, que se sumó al agotamiento que ya existía previamente en el personal de salud.

### Obstáculos y propuestas

Un primer obstáculo que es posible identificar, a lo largo de las entrevistas, está vinculado a la capacitación en investigación en los servicios de salud. Aunque en algunos casos, la revisión científica precede a la revisión ética, los CEI siempre deben tener la oportunidad de combinar ambas revisiones para asegurar el valor social y científico de la investigación^4^. Por consiguiente, deben asegurarse que los estudios propuestos tengan solidez científica y puedan generar información valiosa. En paralelo, deben garantizar que todo el personal de investigación esté capacitado, en virtud de poder llevar adelante con calidad científica la investigación propuesta. En esta dirección, algunos de las personas entrevistadas hicieron referencia a este punto:

*Gente que se mandaba a investigar sin tener ninguna preparación. Muchas veces hacemos, cosa que no está muy bien, algo de docencia, pero es medio complicado porque no podemos ser juez y parte* [...]. *Alguien debería asesorar, sobre todo a los chicos más jóvenes, sobre metodología, básicamente.* (Integrante No. 3 de un CEI)

Sumado a lo anterior, la realización de un proyecto poco sólido desde el punto de vista científico no es ético, en el sentido de que puede exponer a los participantes a riesgos o incomodidades sin ninguna finalidad^14^. Por ende, los CEI deben reconocer que la validez científica de la investigación propuesta es esencial para su aceptabilidad ética. En paralelo, derivado de las entrevistas realizadas surge el siguiente comentario, que identifica otro tipo de falencias formativas en los equipos de salud:

*Sería ideal que exista un curso de ética que, en cualquier momento, uno lo pueda hacer para poder presentar el trabajo. Si el obstáculo para los investigadores es que no hicieron ese curso, tienen que tener a mano un curso que sea gratuito para hacerlo en el momento que puedan. Hay que implementar, de alguna forma, urgente y rápida desde el Central que tengan la formación en ética.* (Integrante No. 2 de un CEI)

De lo anterior se desprende que el entrelazamiento entre lo metodológico y lo ético, en cierta medida, es inevitable. En consecuencia, la división entre capacitación metodológica y evaluación ética se desdibuja en reiteradas oportunidades en la práctica. Una oportunidad de mejora en este punto tiene que ver con la necesidad percibida de espacios de capacitación en ética e investigación en salud específicos para el personal.

Un segundo punto identificado como obstáculo, tiene que ver con lo que en las entrevistas se mencionó como “voluntarismo”. Se ha encontrado, como punto de encuentro entre las experiencias de los distintos CEI, la dimensión de la voluntad de sus participantes como elemento central que da sentido a la tarea a lo largo del tiempo. Como se mencionó anteriormente, en lo relativo al proceso de trabajo y a las dinámicas de organización interna, se subraya la importancia de esta dimensión subjetiva en el funcionamiento.

*El comité específicamente lo que termina siendo es un grupo de voluntarios que tiene mucha voluntad y obviamente uno trata de generar entusiasmo, pero, a veces, esto es todo por voluntad…* (Integrante No. 6 de un CEI)

En esta dirección, y como se expuso en los párrafos anteriores, la falta de estructura puede entenderse como la necesidad de tiempos, recursos, personal específico para la evaluación ética-metodológica, que podría denotar la falta de reconocimiento de la investigación en el campo de la salud pública. En línea con lo anterior, en las entrevistas se destaca la necesidad y la importancia de las estructuras de coordinación y de gestión de políticas de investigación no patrocinada en salud específicas para el sector público. 

Sin embargo, más allá de la dimensión estructural de esta tendencia, es rescatable que, a lo largo de las entrevistas, la idea del voluntarismo que caracteriza la experiencia de trabajo en los CEI también tiene connotaciones positivas.

*Creo que la contraparte del voluntarismo es el impulso subjetivo y colectivo de sostener la tarea. Es decir, la convicción de que esa tarea tiene sentido. La verdad es que varios de los que estamos en el CEI, hacemos investigación, entonces creo que parte del sostenimiento y parte de la convicción tiene que ver con eso también. Con el convencimiento de la importancia de hacer investigación desde el sector salud y que esa investigación esté bien realizada y en los marcos éticos correspondientes. Entonces me parece que, desde el otro lado, pensando desde lo positivo, que a su vez está bueno pensarlo porque si hubiera estructura y no existiera ese convencimiento, tampoco funcionaria, ¿no?* (Integrante No. 1 de un CEI)

Si bien el voluntarismo se menciona como respuesta a la falta de recursos específicos, también representa un elemento que los motoriza. Sin embargo, asumir una estructura organizativa sin considerar la dimensión subjetiva, vinculada a los intereses y trayectorias profesionales de quienes integran los comités, no permite comprender cuáles son las principales motivaciones y el entusiasmo que genera la tarea. En este punto resulta estratégico para la gestión de políticas en salud rescatar y reconocer la existencia de la motivación y el interés de las personas que integran los comités en sostener estos espacios vinculados con la investigación. 

Finalmente, otro elemento que se presenta a lo largo de las entrevistas como un obstáculo se vincula con la revisión del lenguaje en lo relativo al proceso del consentimiento informado. Los efectores públicos de salud de la CABA son los que usualmente brindan cobertura a las poblaciones que se encuentran atravesadas por múltiples vulneraciones de derechos. En este contexto, si bien por un lado la realización de estudios permite la construcción de información para la planificación de intervenciones y la actualización de prácticas sanitarias, por el otro implica una relación asimétrica de poder. De esta forma, el cuidado en el uso del lenguaje resulta un elemento crucial para garantizar las condiciones para obtener el consentimiento informado de quienes sean los sujetos de las investigaciones^19^:

*Muchas veces había cuestiones del consentimiento en relación al lenguaje, un lenguaje no claro. Y era necesario rehacer el consentimiento adecuando el lenguaje a la población destinataria, básicamente eso.* (Integrante No. 1 de un CEI)

La revisión del lenguaje, en el marco de la protección de la confidencialidad de los datos de quienes participan en las investigaciones, es un punto que se menciona en la fundamentación ética del consentimiento informado^19^. Con relación a esto, a lo largo de las entrevistas realizadas se reconocen límites vinculados al uso de las nuevas tecnologías en la investigación en salud y al proceso de consentimiento. El uso de técnicas de *big data* y el procesamiento de grandes volúmenes de datos provenientes de fuentes secundarias se presenta como un fuerte desafío para la labor de los CEI.

*Y, todo eso, está en proceso de transformación… Creo que, en el futuro, vas a tener que capturar a un experto en datos, porque la investigación va hacia ahí. Hay planteos muy serios… Dentro de veinte o treinta años no se van a usar más humanos para probar drogas nuevas; no hace falta exponer a alguien a riesgos porque la información que vas a tener, va a ser suficiente. Eso tiene grandes beneficios y grandes desventajas. Y nosotros no tenemos la capacidad para evaluarlo.* (Integrante No. 5 de un CEI)

Con el proceso de informatización de los servicios de salud públicos en la CABA cobran relevancia, en algunas de las entrevistas realizadas, los obstáculos que se encuentran en la tarea de evaluación ética de proyectos que utilizan métodos y técnicas de *big data* en las investigaciones en salud. Sin embargo, algunas de las consecuencias del uso de este tipo de técnicas informáticas aún son objeto de estudio y discusión. Lecuona nos permite identificar elementos que se traducen en desafíos para el trabajo de los CEI^20^. En esta dirección, se señala la necesidad de equilibrar la protección de la intimidad y la confidencialidad de los datos de las personas usuarias del sistema público de salud y la posibilidad de realizar investigaciones que puedan aprovechar el gran volumen de datos existente en el marco de la sociedad de la información actual. El desafío reside en la capacidad de discernir los borrosos límites entre un problema técnico y uno ético. Para ello, la incorporación de la ciencia de datos y de la capacitación en este campo, se presenta como una oportunidad en la conformación de los CEI como organismos que puedan proteger los derechos de las personas en el contexto actual. 

## CONCLUSIONES

Un primer objetivo que este trabajo se propuso fue desplegar la discusión teórica sobre los resultados parciales alcanzados en el marco de un proyecto de investigación en curso de la Residencia Posbásica de Investigación en Salud de la CABA. Al tratarse de un proyecto que prioriza un enfoque de investigación cualitativa, el trabajo teórico corre en paralelo a la conformación de la muestra^13^. Si bien la posibilidad de generalizar no es una potencialidad de los estudios cualitativos, el trabajo de análisis realizado ha permitido caracterizar escenarios comunes en la práctica de la investigación en los efectores públicos de salud de la CABA en un contexto concreto.

A partir de una estrategia metodológica “flexible”^8^, un segundo objetivo que persiguió este trabajo fue identificar ejes para profundizar el proceso de indagación sobre los sentidos y las prácticas desplegadas a lo largo de las entrevistas. La revisión de las herramientas de relevamiento de datos, principalmente en lo relativo a las entrevistas, resulta un paso fundamental para la selección de nuevas personas a entrevistar. La heterogeneidad y la diversidad de roles se presenta como una posibilidad de seguir identificando sentidos y escenarios comunes en el proceso de implementación cotidiana del marco regulatorio que se presenta en la Ley 3301 de la CABA.

Un primer elemento que queda planteado para ser profundizado en futuras investigaciones es la persistencia de una mayor representatividad de profesiones médicas en el total de miembros integrantes de los CEI en CABA. Si bien a partir de la Ley 3301 se reconoció la importancia de la multidisciplinariedad en relación con la investigación, resulta interesante identificar de qué forma, la investigación en salud como práctica, ha sido apropiada por las otras disciplinas del equipo de salud. Sobre esta base, la incorporación y la efectiva participación del equipo de salud en las tareas de los CEI tiene como condición de posibilidad la socialización y el desarrollo de las habilidades propias de la investigación en salud que no está presente en todas las trayectorias profesionales de igual forma. En esta dirección, la evaluación ética es una tarea compleja que requiere la existencia de distintos perfiles de evaluadores que puedan garantizar una multiplicidad de miradas para asegurar el valor social y científico de una investigación. 

Las problemáticas y situaciones que se abordan como demandas desde el sector salud y, principalmente, desde el subsector público, presentan el desafío para la producción de conocimientos y saberes que permitan reformular las intervenciones y perspectivas existentes. Contar con un equipo de salud que pueda cuestionar los enfoques de la investigación más allá de lo disciplinar, construir nuevos interrogantes y llevar adelante prácticas de investigación situadas, puede pensarse como condición de posibilidad del trabajo interdisciplinario^16^. 

A su vez, un segundo elemento que da lugar a nuevas indagaciones a partir del análisis propuesto se vincula con la participación de la sociedad civil y el rol de la comunidad en la evaluación ética de los proyectos de investigación en salud en el subsector público^16^. Como se ha descrito en este trabajo, actualmente los CEI tienen representantes de la comunidad dentro de sus integrantes. El punto es cómo se operativiza esa representación y participación en los procesos de evaluación.

Un tercer elemento, identificado como escenario recurrente entre los CEI, se vincula con la distribución de las tareas en los procesos de trabajo. Si bien se resalta que esta se organiza, en algunos casos, en función de las trayectorias y disciplinas, la disponibilidad del tiempo es un factor determinante de su funcionamiento. Esta situación subraya las condiciones laborales y el enorme desgaste en el equipo de salud^17^. En esta dirección, se identifica la necesidad de revisar la gestión del tiempo y los recursos como variables críticas en la promoción de la investigación en salud en el subsector público. 

Vinculado a esto, además de garantizar las condiciones de trabajo que permitan y promuevan el desarrollo de la investigación en salud como práctica, es importante recuperar también las condiciones subjetivas señaladas en este trabajo por las personas que integran los CEI. Si bien la disponibilidad del tiempo es una variable crítica, el compromiso con la tarea, la motivación y el interés profesional son elementos claves para la promoción de la investigación en salud. 

Un cuarto elemento pendiente para profundizar, que se desprende del recorrido realizado en este trabajo, señala como área de vacancia el proceso de informatización de los servicios de salud y el impacto en la investigación en salud en el subsector público. La aparición de nuevos enfoques metodológicos, vinculados al uso de técnicas y métodos de *big data* e incluso la utilización de inteligencias artificiales, es un fuerte desafío para la motorización de capacitaciones y el desarrollo de debates éticos^20^.

Finalmente, a catorce años de la implementación de la Ley 3301, este trabajo se propuso identificar obstáculos que pudieran traducirse en oportunidades para la gestión de la investigación en salud pública en la CABA. En este sentido, esperamos que puedan leerse de forma constructiva los puntos que se presentan en este análisis de resultados alcanzados hasta el momento.
